# *ASAP1* gene InDel variants are associated with enhanced goat resistance against *Brucella* infection

**DOI:** 10.5713/ab.250722

**Published:** 2026-03-11

**Authors:** Xiaoyu Liu, Congliang Wang, Xiaoyuan Ren, Zhaofei Ren, Yanyan Li, Wangye Liu, Rongrong Li, Xiaoyue Song, Helin Li, Lei Zhang, Shenghui Chen, Xiaomin Du, Jinlian Hua, Haijing Zhu

**Affiliations:** 1 Shaanxi Provincial Engineering and Technology Research Center of Shaanbei Cashmere Goats, College of Advanced Agricultural Sciences, Yulin University, Yulin, China; 2 College of Veterinary Medicine/Shaanxi Centre of Stem Cells Engineering & Technology, Northwest Agriculture & Forestry University, Yangling, China; 3 Management Committee of Yulin Modern Agricultural Science and Technology Demonstration Zone, Yulin, China; 4 Shaanxi Shenao Agricultural Biotechnology Development Co., Ltd., Yulin, China

**Keywords:** *ASAP1*, *Brucella*, Goats, Resistance

## Abstract

**Objective:**

This study aimed to investigate the potential of the *ASAP1* gene as a genetic biomarker for brucellosis resistance/susceptibility in goats.

**Methods:**

This study collected samples from female Shaanbei white cashmere (SBWC) goats to investigate the association between the *ASAP1* gene and brucellosis susceptibility. Peripheral blood mononuclear cells (PBMCs) were isolated from goats with various haplo types and *Brucella* statuses, and the association was evaluated using polymerase chain re action (PCR), quantitative reverse transcription (qRT)-PCR, and lipopolysaccharide (LPS) stimulation assays.

**Results:**

The *ASAP1* gene was expressed most in the spleen, significantly more than in the kidney and heart (p<0.05). In the SBWC goats population, three genotypes insertion/insertion (II), insertion/deletion (ID), and deletion/deletion (DD) were identified at the P2, P5, and P7 sites of goat *ASAP1* gene. Association analysis showed that P2 and P7 sites variants were associated with host resistance to *Brucella* infection with the II genotype used as reference (p<0.05; p<0.01) and maintained after multiple testing correction. The *ASAP1* gene was observed lower expression in testicular tissues of *Brucella*-infected adult SBWC goats compared to healthy controls (p<0.01). Haplotype analysis revealed Hap3 and Hap5 were associated with brucellosis-resistant compared to Hap1 (p<0.05). PBMCs were isolated from goats carrying Hap1, Hap3, and Hap5. After LPS stimulation, significantly reduced *ASAP1* expression was detected in the susceptible haplotype Hap1 compared to the resistant haplotypes. The highest expression level was exhibited by the most resistant haplotype, Hap5. Furthermore, resistant haplotypes showed more rapid activation of key inflammatory pathways and pro-inflammatory cytokines (*NF-κB, IL-6, TNF-α, IFN-γ*) compared to susceptible Hap1, with faster resolution of the inflammatory response observed, particularly in the most resistant haplotype Hap5.

**Conclusion:**

The present study demonstrates that *ASAP1* gene InDel variants influence brucellosis resistance in SBWC goats, providing a theoretical basis for breeding resistant populations.

## INTRODUCTION

Brucellosis, which is by *Brucella* infection, is one of the most important and widespread bacterial zoonotic diseases worldwide. *Brucella* is a Gram-negative, short rod-shaped bacterium. Currently, six species and 19 biotypes have been identified. Among which *Brucella melitensis* [1] and *Brucella abortus* [2] pose the most significant threats to human health. In female animals, *Brucella* infected can cause abortion, loss of appetite, and difficulty walking, while male animals primarily develop orchitis and epididymitis [3].

According to an analysis of global and regional high-risk populations, approximately 500,000 human cases occur worldwide each year, with under-developed regions in Africa and Asia bearing the brunt of the burden [4]. Previous studies have shown that the host resistance to infectious disease is closely linked to genetic factors [5]. Therefore, identifying genetic markers associated with reduced risk of *Brucella* infection in goats and expanding disease-resistant populations represnt a fundamental strategy for mitigating the impacts of brucellosis. With rapid advances in genetics, molecular marker-assisted selection (MAS) is now widely used to improve growth and reproductive performance in goats, Among the available markers, SNP and InDels are the most commonly used [6], as they enable the accurate and rapidly identification of superior genotypes in livestock [7].

The ArfGAP with SH3 domain, ankyrin repeat and PH domain 1 (*ASAP1*) gene produces a protein that contains SH3, ANK, and PH domains. This protein participates in cellular processes such as cytoskeletal regulation and vesicle transport [8,9]. *ASAP1* has been show to regulate the phagocytic capacity of THP-1–derived macrophages against *Mycobacterium tuberculosis* H37Ra by remodeling the actin cytoskeleton [10]. The *ASAP1* protein is predominantly expressed in specific immune cells, such as dendritic cells (DCs). Notably, infection with *M.tuberculosis* significantly downregulates *ASA*P*1* expression in DCs [11]. However, the unique structure of *Brucella* lipopolysaccharide (LPS) distinguishes it from that of typical Gram-negative bacteria like *Escherichia coli*. Specifically, the lipid A moiety of *Brucella* LPS has a significantly longer fatty acid chain. This structural difference alters the molecule conformation and impairs its ability to bind effectively to the toll-like receptor 4/myeloid differentiation factor 2 (TLR4/MD-2) complex. Consequently, *Brucella* LPS induces *NF-κB* mediated immune cell activation to a mch lesser extent than *E. coli* LPS [12]. This failure to trigger a robust inflammatory response in DCs, a form of molecular mimicry may help *Brucella* evade detection by the host’s innate immune system.

To date, no studies have linked the *ASAP1* gene to resistance against *Brucella* infection in goats. In this study, we first analyzed the expression profile of the *ASAP1* gene in various tissues of Shaanbei white cashmere (SBWC) goats. We then detecte polymorphisms at InDel loci within the *ASAP1* gene in goat populations and systematically invesrigated the correlation between these InDel variations and resistance to *Brucella* in SBWC goats.

## MATERIALS AND METHODS

### Experimental animals and sample collection

All experimental animals were obtained from a SBWC goats farm in Yulin City, Shaanxi Province, China. The goats were raised under identical feeding management and environmental conditions, including a standardized diet (comprising a total mixed ration of hay, corn silage, and concentrated feed), a controlled ambient temperature of 15°C–25°C, relative humidity of 50%–70%, a natural light cycle, and a set of biosecurity practices (including isolation of newly introduced animals, scheduled vaccination and deworming, regular disinfection of enclosures, and rodent control). These measures ensured a comparable brucellosis infection risk across all selected samples. A total of 1,145 unrelated adult female SBWC goats were randomly selected based on the following criteria: all goats were 3–5 years old, had a parity of 2–5, Although some goats were infected with *Brucella*, all exhibited a subclinical infection status, showing no abnormalities observed in mental status, appetite, or other clinical signs. 2 mL blood samples were collected via jugular venipuncture. The Rose Bengal Plate Agglutination Test (RBPT) was employed for serological testing of serum samples. Approximately 100 mg of ear tissue samples were collected and stored in 75% ethanol for DNA extraction. Various tissue samples, including major organs (heart, liver, spleen, lung, kidney), digestive tracts (rumen, small and large intestines), neural systems (cerebrum, cerebellum), and other tissues (skin, muscle, fat, ovary) were collected from healthy adult female SBWC goats. Testicular tissues were also obtained from 3-year-old male goats. Additionally, testicular tissue samples were collected from *Brucella* infected 3-year-old SBWC buck under the supervision of epidemic prevention authorities; these sample were aseptically sealed and stored. All tissue samples were kept at −80°C for subsequent RNA extraction.

### Rose bengal plate test

After serum was separated from the collected blood, 20 μL was aliquoted onto the test plate. An equal volume of antigen was then added and thoroughly mixed with the test sample. The mixture was incubated at room temperature (approximately 20°C–25°C) for 4 min. The results was compared with standard positive and negative controls. Samples were recorded as positive if agglutination was observed, and negative if no agglutination was detected. All positive samples were re-examined to confirm the results. Subesequent analysis confirmed that among the 1,145 collected samples, 450 tested positive and 695 tested negative.

### Primer design

The reference sequence of the goat *ASAP1* gene (GenBank accession no. NC_030821.1) was acquired from the NCBI database (https://www.ncbi.nlm.nih.gov; accessed on 5 January 2024). Primers for the amplification of partial fragments of the *ASAP1*, *GAPDH, NF-κB*, and *IL-6*, *TNF-α*, *IFN-γ*, *TGF-β* genes were designed using the online NCBI Primer-BLAST tool (https://blast.ncbi.nlm.nih.gov; accessed on 17 December 2024). InDel variant information was screened and obtained from the Ensembl database (https://asia.ensembl.org/index.html) (Table 1).

### RNA extraction and quantitative reverse transcription-polymerase chain reaction

Total RNA was isolated from collected tissue samples using TRIzol total RNA extraction reagent (Takara), in conjunction with isopropanol, anhydrous ethanol, chloroform, and other associated reagents. The first strand of cDNA was synthesized using the Prime Script RT kit (Takara). The resulting cDNA was diluted to a concentration for gene tissue expression analysis.

Quantitative reverse transcription-polymerase chain reaction (qRT-PCR) was performed using a 20 μL system, comprising 10 μL of 2×ChamQ SYBR qPCR Master Mix, 8 μL of RNase-free ddH_2_O, 1 μL each of upstream and downstream primers, and 1 μL cDNA template. The qRT-PCR amplification was conducted using a three-step program: pre-denaturation at 95°C for 3 minutes; followed by 40 cycles of denaturation at 95°C for 10 seconds and annealing/extension at 55°C for 30 seconds. The *GAPDH* geneserved as the internal reference, and the relative expression levels in each tissue were calculated using the 2^−ΔΔCT^ method. All cDNA samples from different tissues were analyzed with three technical repeates [13].

### DNA extraction and polymorphism detection

Genomic DNA was extracted from collected tissue samples using the high-salt method, The purity and concentration of each sample were measured using a NanoDrop 2000 spectrophotometer (Thermo Fisher Scientific). Finally, qualified DNA samples were diluted to a uniform concentration of 20 ng/μL and stored at −20°C.

The PCR reaction was carried out in a 13 μL system. The reaction procedure was composed of pre-denaturation at 95°C for 5 minutes, followed by 35 cycles of denaturation at 95°C for 1 minutes, annealing at 50°C–65°C for 30 seconds, and extension at 72°C for 30 seconds; with a final extension step at 72°C for 7 minutes. The amplified products werethen held at 4°C. The PCR products were immediately analyzed by 3% agarose gel electrophoresis for genotyping. Sequencing was performed by a commercial provider (Sangon Biotech).

### Lipopolysaccharide stimulation of goat peripheral blood mononuclear cells and cytokine detection

Goats whose genotype distribution matched Hap1, Hap3, and Hap5 of the goat *ASAP1* gene were selected with three individuals from each haplotype serving as biological replicates. After the selected goats were confirmed negative by the RBPT, peripheral blood samples were collected. Goat peripheral blood mononuclear cells (PBMCs) were isolated using a commercial kit (TBD). After cells were cultured for 48 h in RPMI-1640 medium (Gibco) containing 10% fetal bovine serum and 1% dual antibiotics, the supernatant was removed, and any non-adherent or dead cells were rinsed away with PBS. The medium was replaced with fresh medium containing 500 ng/mL *B. melitensis* derived LPS, and the cultivation was continued for 24 h. Samples were harvested at 0, 1, 3, 6, 12, and 24 h for RNA extraction. The first strand of cDNA was synthesized to detect the expression levels of inflammatory factors, including *NF-κB*, *IL-6*, *TGF-β*, *IFN-γ*, *TNF-α* and *ASAP1*, with *GAPDH* serving as the internal reference gene. The relative expression levels of inflammatory factors were analyzed using the 2^−ΔΔCT^ method, with each sample assayed in triplicate.

### Statistical analysis

qRT-PCR results were analyzed using GraphPad prism ver. 10.5.0. Genetic diversity parameters for the *ASAP1* gene variant loci, including population heterozygosity (He), homozygosity (Ho), and polymorphism information content (PIC), were estimated based on the Nei method [14]. The Hardy-Weinberg equilibrium (HWE) of these variant loci was assessed using the SHEsis (https://github.com/celaoforever/SHEsisPlus/blob/master/README.md ; accessed on 23 July 2024) platform and the Gdicall (http://www.msrcall.com/Gdicall.aspx; accessed on 23 July 2024) website. Differences in the distribution frequencies of genotypes and alleles were evaluated by the chi-squared test in SPSS 26.0 software. A logistic regression model was constructed to calculate odds ratios (OR) and 95% confidence intervals (95% CI) for estimating the association strength between genetic variations and phenotypes under different genetic models (codominant, dominant, recessive, and allele models). Additionally, linkage disequilibrium (LD) analysis was conducted, and haplotypes were inferred. LD indicates non-random associations between different loci, while haplotype construction reveals the combination patterns of gene variations and their distribution with the population.

### Bioinformatics analysis

To investigate the impact of *ASAP1* gene InDel variation sites on transcriptional activity, the AliBaba 2.1 website (http://gene-regulation.com/pub/programs/alibaba2/; accessed on 25 June 2024) was used to predict transcription factor binding sties within the intronic region harboring the InDel, and the differential transcription factors were marked with red triangles.

Nucleotide and protein sequences for six species (*Capra hircus*, *Ovis aries*, *Bos taurus*, *Sus scrofa*, *Gallus gallus*, and *Homo sapiens*) were obtained from the NCBI database (https://www.ncbi.nlm.nih.gov/; accessed on 27 July 2024). The MegAlign software ver. 7.2.0 was used to compare nucleotide sequence homology and the MEGA11 ver. 11 software (University Park) was used to construct a phylogenetic tree.

## RESULTS

### Expression levels of *ASAP1* in testicular tissues of buck Shaanbei white cashmere goats infected/uninfected with *Brucella*

**Figure 1:**
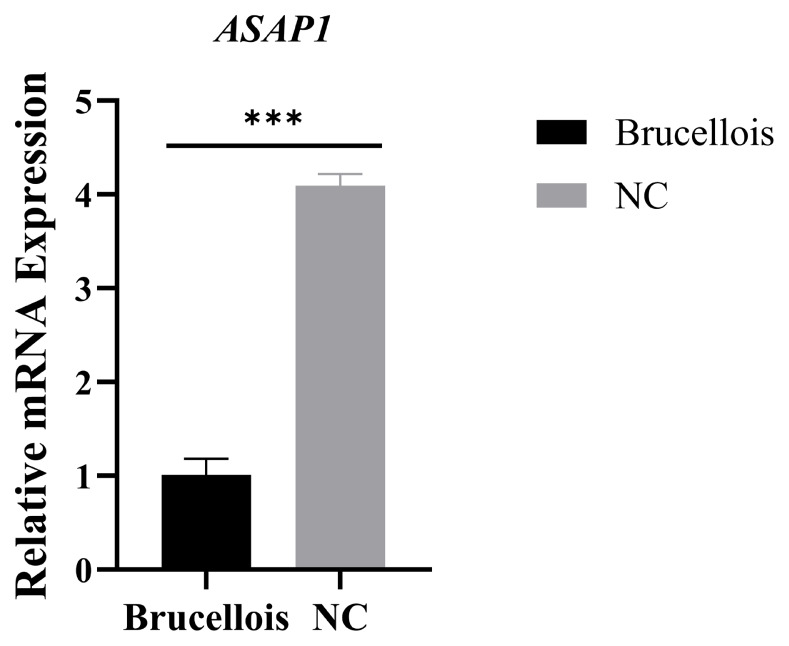
Expression of *ASAP1* in testicular tissues from 3-year-old male Shaanbei white cashmere goats with or without *Brucella* infection. Gene expression was quantified using the 2^−ΔΔCT^ method normalized to *GAPDH*. Data are presented as mean±SEM (n = 3 biological replicates per group). NC indicates negative control; *** indicates p<0.001 by t-test. SEM, standard error of the mean.

Testicular tissue samples were obtained from a 3-year-old *Brucella* positive buck and a healthy buck of the same age and breed. Quantitative analysis demonstrated that the expression level of the *ASAP1* gene was significantly lower in the *Brucella* infected buck compared to the healthy control (p<0.01) (Figure 1).

### Expression levels of the *ASAP1* gene in various tissues of Shaanbei white cashmere goats

**Figure 2:**
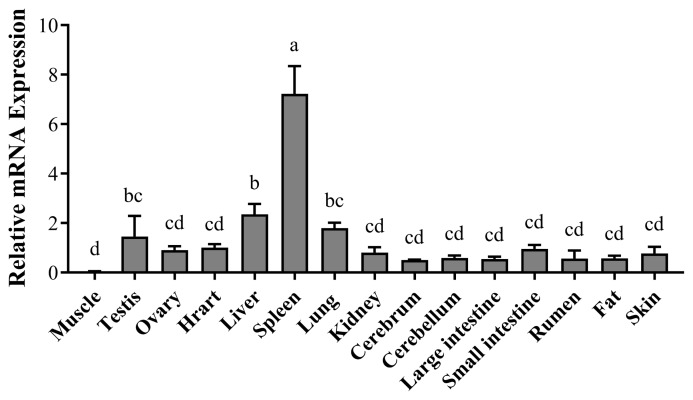
Tissue expression profile of the *ASAP1* gene in healthy adult female goats. Gene expression was quantified using the 2^−ΔΔCT^ method normalized to *GAPDH*. Values represent mean±SEM of three biological replicates. ^a–d^ Statistically significant differences (p<0.05) determined by one-way ANOVA with Tukey’s post hoc test are indicated by different letters. SEM, standard error of the mean.

qRT-PCR results showed that the *ASAP1* gene was expressed in all examined tissues of SBWC goats. The highest expression level was detected in the spleen, which was significantly higher than that in all other tissues (p*<*0.05) (Figure 2).

### Identification of *ASAP1* gene insertion/deletion variants

**Figure 3:**
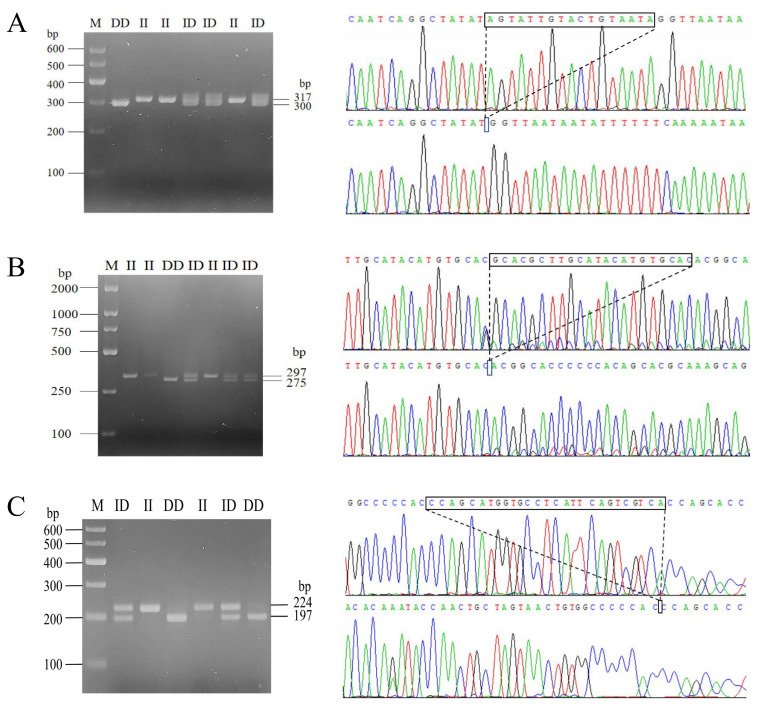
Electrophoresis and sequencing diagrams of the goat *ASAP1* gene InDel. (A) Electrophoresis and sequencing diagrams of the P2 mutation site. (B) Electrophoresis and sequencing diagrams of the P5 mutation site. (C) Electrophoresis and sequencing diagrams of the P7 mutation site. M: 600 bp marker (A, C); 2,000 bp marker (B); II, insertion/insertion, ID, insertion/deletion; DD, deletion/deletion.

Following verification by 3% agarose gel electrophoresis and sequencing, polymorphism was confirmed in three out of the ten selected *ASAP1* gene intronic loci P2, P5 and P7 (Figure 3). Three genotypes insertion/insertion (II), insertion/deletion (ID), and deletion/deletion (DD) were identified at each of the variant loci P2 (Figure 3A), P5 (Figure 3B), and P7 (Figure 3C). The band sizes for the P2 locus corresponded to 317 bp (II), 317 bp and 300 bp (ID), and 300 bp (DD). For the P5 locus, the band sizes corresponded to 297 bp (II), 297 bp and 275 bp (ID), and 275 bp (DD). The P7 locus showed band sizes of 224 bp (II), 224 bp and 197 bp (ID), and 197 bp (DD). Notably, the sequences of the P2 and P5 loci differed from the predicted variant sequences in the Ensembl database. The InDel sequence obtained through actual sequencing was /AGTATTGTACTGTAATA which differed from the prediction for the P2 locus (TCGTCTGCGACGTGG). The InDel sequence of P5 locus obtained from actual result was GCAC GCTTGCATACATGTGCAC, which also differed from the prediction (TGCACATGTATGCAAGCGTGCC). In contrast, the sequence of P7 InDel locus matched the predicted sequence in the Ensembl database (Figure 3C).

### Genetic parameter analysis of goat *ASAP1* gene InDel

The genetic parameter results for the P2, P5 and P7 variant sites of the *ASAP1* gene in SBWC goats are presented in Table 2. A total of 1133, 1054, and 1090 goat genomic samples were successfully genotyped at the P2, P5 and P7 loci, respectively. At the P2 and P7 loci, the frequency of the “I” allele was higher than that of the “D” allele, while the opposite pattern was observed at the P5 locus. Based on the PIC values, the P5 and P7 loci showed moderate polymorphism (0.25<PIC<0.50). In contrast, the P2 locus displayed moderate polymorphism (0.25<PIC<0.50) only in the case group, while it showed low polymorphism (PIC<0.25) in both the control group and the overall population. Additionally, the P2 locus conformed to HWE in the case group and the total population (p*>*0.05), but deviated from HWE in the control group (p*<*0.05). For the P5 and P7 loci, all groups conformed to HWE (p*>*0.05), with the exception of the P7 locus in the total population, which deviated significantly (p*<*0.05).

### Distribution of genotypes and alleles at different *ASAP1* gene variants in cases and controls

The genotypes and allele frequency distributions of the three *ASAP1* gene varient sites in goats were analyzed statically. The result showed that the distribution frequencies of the three genotypes differed significantly between the case and control groups at the P2 and P7 sites (p*<*0.05), whereas no significant difference was observed for allele (p*>*0.05). At the P5 locus, neither the genotype nor allele distribution frequencies showed significant differences (p*>*0.05) (Table 3). These findings suggest that the P2 and P7 variant loci may be associated with resistance to *Brucella* infection.

### Age and parity as potential risk factors for *Brucella* infection in goats

To exclude potential confounding effects of age and parity on brucellosis risk, association analyses between these factors and *Brucella* infection were conducted. The analysis confirmed that neither age (Table 4) nor parity (Table 5) was significantly associated with *Brucella* infection risk in the studied population (p>0.05). Nevertheless, these non-significant variables were still incorporated into the final logistic regression models to ensure control for potential confounding effects and to improve the accuracy of the estimated associations between *ASAP1* polymorphisms and infection status.

### Association analysis between *ASAP1* gene polymorphism and resistance against brucellosis in goats

To investigate the association between the *ASAP1* gene and resistance to brucellosis in goats, 4 genetic models (codominant, dominant, recessive, and allelic) were constructed. Logistic regression analysis was performed to evaluate the association of the P2, P5 and P7 loci with resistance. The results demonstrated that both the codominant (ID vs II) and dominant (ID+DD vs II) models at the P2 locus were significantly associated with a reduced risk of brucellosis when the II genotype was used as the reference. The OR were 0.723 (p = 0.017, 95% CI: 0.553–0.944) and 0.769 (p = 0.045, 95% CI: 0.595–0.994), respectively, suggesting that the II genotype confers susceptibility. In contrast, at the P7 locus, the codominant (ID vs II) and dominant (ID+DD vs II) models were significantly associated with an increased risk of brucellosis, with OR of 1.559 (p = 0.001, 95% CI: 1.210–2.008) and 1.462 (p = 0.050, 95% CI: 1.147–1.864), respectively. This indicates that the II genotype is associated with resistance. Furthermore, the p values from logistic regression models for the three InDel polymorphisms under different genetic models were corrected using the false discovery rate (FDR) method. The results indicated that the codominant models of loci P2 and P7, along with the dominant model of locus P7, remained statistically significant after FDR correction (FDR adjusted p<0.05) (Table 6).

### Linkage disequilibrium and haplotype association analysis of the InDel locus in goat *ASAP1* gene

**Figure 4:**
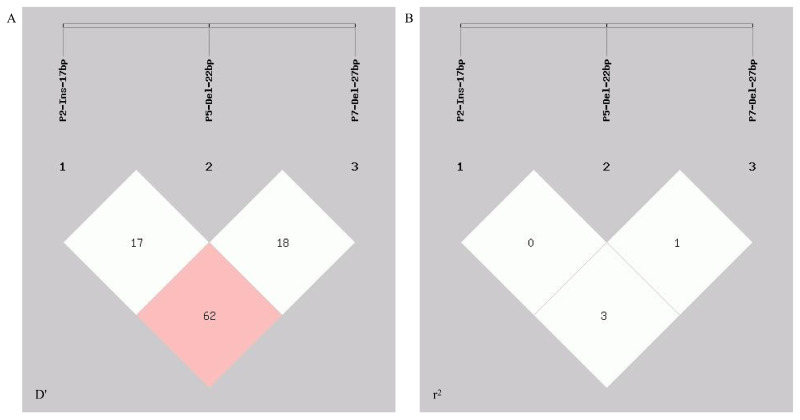
LD between P2, P5, and P7 variant sites in the goat *ASAP1* gene. (A) D′ value, (B) r2 value. LD, linkage disequilibrium.

Analysis of the LD relationship among the three variant sites of the *ASAP1* gene was conducted on the Gdicall website. The D′ and r^2^ values between sites P2 and P5 were 0.179 and 0.007, respectively; between P2 and P7, the values were 0.185 and 0.016, respectively; and between P5 and P7, the values were 0.620 and 0.036, respectively. These results indicate that no strong linkage exists among the variant sites P2, P5, and P7 (Figure 4).

Although the three loci were not in strong LD, haplotype analysis was still performed, supported by our gene expression date and based on the hypothesis of their potential functional complementarity in regulating *ASAP1* expression. Using the P2, P5, and P7 variant sites, 8 haplotypes were constructed: I_P2_I_P5_I_P7_, I_P2_I_P5_D_P7_, I_P2_D_P5_I_P7_, I_P2_D_P5_D_P7_, D_P2_I_P5_I_P7_, D_P2_D_P5_I_P7_, D_P2_I_P5_D_P7_, and D_P2_D_P5_D_P7_. They analysis showed that the risks of brucellosis for haplotypes I_P2_D_P5_I_P7_ and D_P2_I_P5_I_P7_ were 0.761 and 0.657 times that of the I_P2_I_P5_I_P7_ haplotype (p = 0.035, 95% CI = 0.590–0.980; p = 0.016, 95% CI = 0.467–0.924), suggesting that I_P2_I_P5_I_P7_ is a susceptibility haplotype (Table 7).

### Changes in cytokine mRNA expression levels in peripheral blood mononuclear cells stimulated by lipopolysaccharide *in vitro*

**Figure 5:**
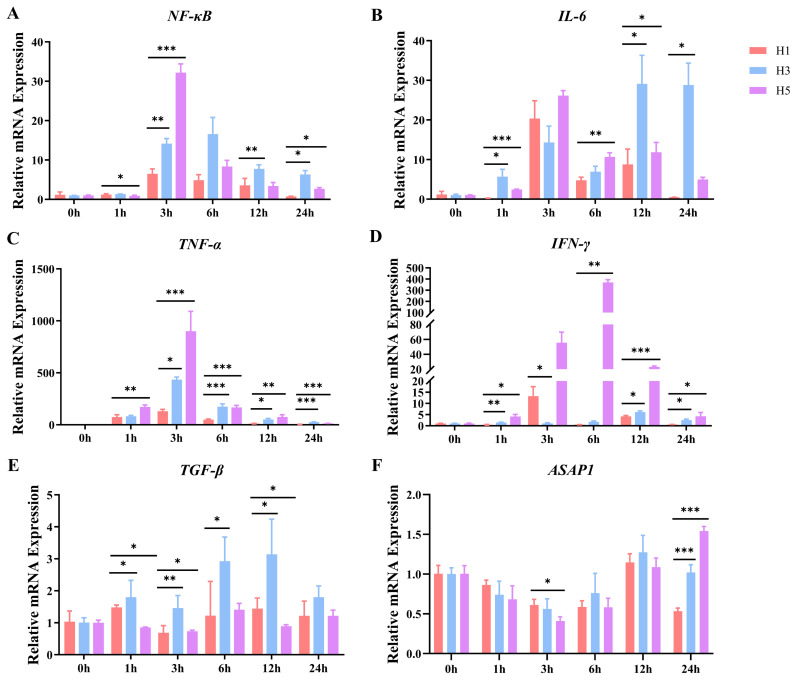
Changes in cytokine expression in PBMCs from goats of different genotypes following LPS stimulation. Gene expression was quantified using the 2^−ΔΔCT^ method normalized to *GAPDH*. (A–F) show the changes in the expression levels of cytokines such as *NF-κB*, *IL-6*, *TNF-α*, *IFN-γ*, *TGF-β* and *ASAP1* at different time points after LPS stimulation across various haplotypes. n = 3; * p<0.05; ** p<0.01; *** p<0.001. PBMCs, peripheral blood mononuclear cells; LPS, lipopolysaccharide.

Based on previous analysis findings in which Hap3 and Hap5 were associated with a significantly reduced risk of brucellosis in goats compared to Hap1 (p*<*0.05), the dynamic expression of cytokines in PBMCs/macrophages from goats with different haplotypes was further examined following LPS stimulation (Figure 5). The results showed that at 3 h and 24 h post-LPS stimulation, *NF-κB* expression in PBMCs from Hap3 and Hap5 was significantly higher than that in Hap1 (p*<*0.05) (Figure 5A), with similar trends observed at other time points. At 6 h and 12 h after LPS stimulation, *IL-6* expression levels in Hap5 were significantly higher than those in Hap1 (p*<*0.05). Similarly, at 12 h and 24 h post-stimulation, *IL-6* expression in Hap3 was also significantly elevated compared to Hap1 (p*<* 0.05) (Figure 5B). Throughout the 3–24 h period following LPS stimulation, *TNF-α* expression levels in PBMCs from Hap3 and Hap5 were consistently significantly higher than those in Hap1 (p*<*0.05) (Figure 5C). Furthermore, at 12 h and 24 h after LPS stimulation, significantly greater secretion of *IFN-γ* was observed in PBMCs from Hap3 and Hap5 (p*<*0.05) (Figure 5D). Additionally, *TGF-β* expression in PBMCs from Hap3 was significantly higher than in Hap1 from 1 h to 12 h after LPS stimulation (Figure 5E). Interestingly, LPS stimulation induced a consistently weaker inflammatory response in the susceptible Hap1 than in the resistant haplotypes (Hap3 and Hap5). Notably, after 6 h, the levels of inflammatory cytokines in Hap5 were, on average, lower than those in Hap3. This pattern suggests that the susceptible Hap1 haplotype mounts a diminished response to bacterial challenge, whereas the resistant haplotypes mount robust responses. Moreover, the most protective haplotype, Hap5, not only mounts a stronger inflammatory response but also resolves inflammation more rapidly, thus facilitating quicker restoration of immunological homeostasis. Following 24 h of LPS stimulation, the expression levels of *ASAP1* PBMCs from Hap5 and Hap3 were significantly higher than those in Hap1 (Figure 5F). This observation is further supported by the similar expression pattern of *ASAP1* in *Brucella* infected and uninfected testicular tissues.

### Transcription factor binding prediction

**Figure 6:**
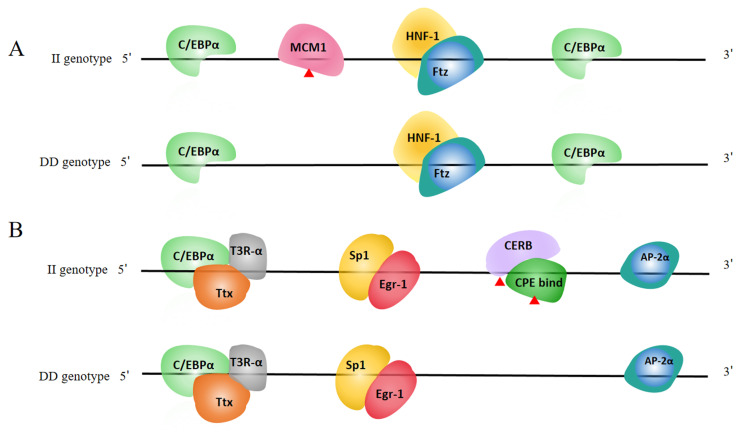
Predicted transcription factor binding sites at the P2 and P7 mutation sites of the goat *ASAP1* gene. (A) Predicted differential transcription factors for the II and DD genotypes at the P2 locus: *MCM1*. (B) Predicted differential transcription factors for the II and DD genotypes at the P7 locus: *CREB* and *CPEbind*. Differential transcription factors are indicated by red triangles below. II, insertion/insertion; DD, deletion/deletion.

Prediction of transcription factor binding to the variant sites was performed (Supplement 1), the analysis revealed that, compared to the deleted sequence, the inserted sequence at the P2 site specifically bound the transcription factor *MCM1* (Figure 6A; Supplement 2A), while the inserted sequence at the P7 site was specifically bound by the cAMP response element-binding protein (*CREB*) and the cytoplasmic polyadenylation element-binding protein (*CPEB*) (Figure 6B; Supplement 2B).

### Gene conservation analysis

The results of MegAlign alignment for species homology showed that the nucleotide sequence of the goat *ASAP1* gene exhibited the highest homology with *Ovis aries* (98.7%) and *Bos taurus* (96.3%), and the lowest homology with *Homo sapiens* (86.4%) and *Gallus gallus* (71.4%) (Supplement 3A). A phylogenetic tree of the protein was constructed using MEGA11 software, which revealed that the genetic distance between the goat *ASAP1* gene and *Bos taurus* was the closest (Supplement 3B).

## DISCUSSION

*Brucella*, a facultative intracellular Gram-negative bacterium, invades immune cells such as macrophages, thereby triggering a strong inflammatory response in the host. The *ASAP1* gene functions to remodel the cytoskeleton by enhancing F-actin aggregation and increasing the formation of vinculin/paxillin plaques, thereby altering actin remodeling dynamics [10]. Evidence indicates that the endocytosis of *M.tuberculosis* H37Ra in THP-1 macrophages is achieved through the regulation of actin dynamics by the *ASAP1* gene [10]. Notably, both *M.tuberculosis* and *Brucella* are intracellular bacterias that evade immune clearance and achieve intracellular proliferation through common mechanisms such as inhibiting phagosome-lysosome fusion and modulating autophagy pathways [15]. Genome-wide association studies have established that specific SNPs in the *ASAP1* gene are significantly linked to human susceptibility to tuberculosis. In addition, *ASAP1* expression was downregulated and DC migration was severely impaired following infection with *M.tuberculosis* [11], which prevented the effective activation of early T-cells and lead to a failure in establishing an adaptive immune response [16]. This has been identified as a key mechanism for host susceptibility to tuberculosis. Importantly consistent with findings in tuberculosis [11], our study found a significantly lower expression level of the *ASAP1* gene in the testicular tissues of *Brucella* infected adult bucks compared to healthy controls. It has been reported that during *Brucella* infection, the effector protein BspF interacts with an Arf6 GTPase-activating protein (*ACAP1*), interfering with *Arf6/Rab8a* mediated membrane trafficking and causing abnormal accumulation of trans-golgi network (TGN) derived vesicles on the bacterium-containing vacuole (rBCV), thereby promoting bacterial replication [17]. *ASAP1* and *ACAP1* belong to the Arf-GAP protein family [17], but *ASAP1* is uniquely characterized by an SH3 domain [18], which mediates protein-protein interaction networks by recognizing proline-rich motifs [19], and is widely involved in processes such as cell migration, proliferation, and cytoskeletal remodeling [19,20]. Although the functional importance of *ASAP1* had been demonstrated in colorectal cancer [21], gastric cancer [20], and tuberculosis [11], its role in *Brucella* infection had not been reported.

This study represents the first report to systematically investigate the association between *ASAP1* gene variations and *Brucella* infection risk in goats. The expression of *ASAP1* was first analyzed across various goat tissues, This expression profile suggested a potential association of this gene with immune function in animals [22]. As a vital immune organ, the spleen is recognized as a primary defense against bacterial infections through the synergistic actions of innate immunity, adaptive immunity, and mechanical filtration [23]. Therefore, the high expression of the *ASAP1* gene in this organ likely indicated a significant role in immune responses and pathogen clearance in goats. Evolutionary genetic analyses have indicated that positive selection pressure on host genes is closely associated with pathogen-driven adaptive evolution [24]. In the SBWC goat population, three InDel loci within the *ASAP1* gene were found to display significant population genetic characteristics. We analyzed the genetic diversity and HWE of thise loci, among them, the P2 and P7 loci were predominantly characterized by the I allele, and distinct genotype distributions were observed between populations. The observed deviation from HWE at the control group in the P2 locus could be attributed to breeding practices intrinsic to commercial livestock management. Directed selection for economically important traits and non-random mating schemes (e.g., the extensive use of elite bucks) will systematically alter genotype frequencies, leading to deviations from HWE expectations [25]. Despite these population genetic deviations, the observed associations between *ASAP1* InDels and brucellosis susceptibility were further supported by functional evidence from gene expression analyses.

Given that previous studies have often been limited by small sample sizes (N<500), this study provides reliable evidence for the association between *ASAP1* gene polymorphisms and resistance/susceptibility of goats to *Brucella* infection through a large sample population (N>1,000). Due to the large sample size and associated cost constraints, confirmatory ELISA or PCR testing was not performed. However, it is acknowledged that the sole reliance on the RBPT for infection status classification constitutes a methodological limitation. Given that results based on RBPT do not exclude the possibility of false negatives or false positives, to improve reliability in the absence of confirmatory assays, all samples underwent repeated RBPT testing, and only those with consistently positive results were included in the infected group, which has substantially reduced the probability of false positives within the constraints of the available methodology. Prior to the genetic association analysis, the potential associations of age and parity with *Brucella* infection status were assessed in the selected samples. The confirmation of no significant associations for these covariates strengthened the validity of the genotypic results. It is acknowledged that environmental and management variables, including pen allocation and microenvironment, were not controlled for in this study. Nevertheless, all experimental animals were sourced from farms with standardized management, and the association between *ASAP1* genotypes and infection status remained significant after adjusting for age and parity. The association analysis revealed that genotype II at the P2 locus was associated with susceptibility, whereas genotype II at the P7 locus was associated with resistance, suggesting that genotype distribution influences the response of goats to *Brucella* infection. Previous studies have indicated significant associations between genetic polymorphisms and increased or decreased to *Brucella* infection. In our earlier work, the cytotoxic T lymphocyte-associated antigen-4 (*CTLA4*) II genotype was shown to reduce brucellosis susceptibility in goats and enhance secretion of anti-inflammatory factors from PBMCs post-LPS challenge [26]. In our study, although no LD was detected among the three InDel loci, based on previous research in regulatory genomics, it has been proposed that combinations of genetic variants, even in the absence of strong linkage, could encompass multiple cis-regulatory modules (e.g., enhancers or silencers) and that their joint effects might co-regulate target gene expression through mechanisms such as chromatin spatial reorganization [27]. Furthermore, studies suggest that such combinatorial variation may influence transcription-factor networks and protein interactions, thereby potentially contributing to complex phenotypes like disease resistance in a synergistic manner [28]. Accordingly, we continue to explore the non-linked haplotype model from functional and regulatory perspectives. Therefore, PBMCs from goats with different haplotypes were stimulated with LPS. Observations suggested that PBMCs from goats carrying the Hap3 and Hap5 haplotypes tended to exhibit a more rapid initiation and resolution of the immune response following stimulation. These changes appeared to involve activation of the *NF-κB* pathway, relatively elevated expression levels of pro-inflammatory cytokines (*IL-6* and *TNF-α*), and increased secretion of immunoregulatory factors (*TGF-β* and *IFN-γ*). At 24 h post-LPS stimulation, the *NF-κB* pathway showed indications of downregulation, and the concentrations of pro-inflammatory and immunoregulatory factors decreased. This response profile could potentially contribute to more effective control of infection. Furthermore, the expression level of *ASAP1* at 24 h was significantly lower in haplotypes associated with high infection risk compared to those associated with low infection risk, which aligns with the downregulated expression trend of *ASAP1* observed in infected animals. In *Brucella* infected testicular tissue expression was observed aligning directionally with our cellular assay results. However, given that the sample size was limited to n = 1 per group, this observation should be considered preliminary and does not constitute definitive evidence regarding the role of *ASAP1* in goat *Brucella* infection. And the testicular tissue examined in this study serves as a primary reproductive organ target for *Brucella* infection, offering relevant pathophysiological insights. It should be noted, however, that sample collection from infected animals was highly restricted, and all animals in the main association cohort were female. Therefore, findings from this male tissue analysis should be interpreted as a reference for potential infection effects in reproductive organs. Overall, these three gene loci are genetically independent; based on our exploratory observations, they might collectively influence the immune phenotype when co-present. Nevertheless, the precise mechanisms underlying these phenotypic associations warrant further investigation.

Although the protein-coding region directly determined protein structure, gene expression can be regulated by mutations in non-coding regions through alteration of transcription factor binding affinity, a process that plays a significant role in the evolution of disease resistance traits [29]. Previous studies had shown that intron 3 of the mediator of IRF3 activation (*MITA*) gene was bound by the RNA binding protein LUC7L2, which leads to intron retention and the subsequent triggers nonsense-mediated mRNA decay (NMD), thereby reducing *MITA* protein levels and weakening the intensity of the innate immune response to DNA viruses [30]. Importantly, transcription factors including *MCM1*, *CREB*, and *CPEB* play crucial roles in regulating the immune cell cycle, immune gene expression, and cell migration [31–33]. It should be emphasized that the transcription-factor binding sites (TFBS) predicted using AliBaba2 are based solely on in silico sequence analysis; these predictions are preliminary and do not confirm actual regulatory function, their specific mechanisms require further investigation. Considering that cattle, sheep, and humans are all major hosts of *Brucella*, and given the high expression of *ASAP1* was observed in goat spleen in this study, a cross-species homology comparison was conducted. Phylogenetic analysis showed that the goat *ASAP1* nucleotide sequence is highly conserved with *Ovis aries* (98.7%) and *Bos taurus* (96.3%), but less so with *Gallus gallus* (71.4%), indicating strong evolutionary conservation among ruminants, as reported in existing comparative genomic studies [34]. In summary, InDel variant in the *ASAP1* gene were found to be associated with resistance to *Brucella* infection in goats, Goats carrying the low-disease-risk haplotype were shown to initiate immune responses more rapidly to combat bacterial infection and clear the pathogen. This outcome may be attributed either to potential functional interactions among the three variant sites or to the differential recruitment of transcription factors (such as *MCM1*), both of which could ultimately regulate gene function. However, given the high complexity of bacterial invasion and clearance, the precise molecular mechanisms were concluded to require further investigation.

## CONCLUSION

Polymorphisms at three InDel loci within the *ASAP1* gene were identified for the first time in the SBWC goat population, and their association with increased or decreased to *Brucella* infection was established. Under LPS stimulation, PBMCs from goats carrying the low-disease-risk haplotype exhibited a robust immune response and an accelerated termination of the immune response. The high-disease-risk haplotype exhibited lower *ASAP1* levels, a pattern that matches the expression trend observed in tissues from *Brucella*-infected goats. This study elucidates the role of the *ASAP1* gene in resistance to *Brucella* infection and provides potential target loci for the genetic breeding of brucellosis-resistant goats.

## Figures and Tables

**Table 1:** Goat *ASAP1* gene InDel detection and qRT-PCR primer pairs

Primer	Information	Primer sequence (5′–3′)	Length (bp)	Tm (°C)	Function
P1	rs642042901 chr14:71401052–71401053	F: GCCTTAGCCTTGAGTAGCCC R: AAACAGCACAGCATCCCAGT	294/318	60	Indel detection
P2	rs655531471 chr14:71407951–71407952	F: TTTGAAAACTTCGGGCCGC R: TGGGGACGACAACATAAAGCA	300/317	59	Indel detection
P3	rs672114848 chr14:71396371–71396396	F: CTCACGGTTTCTGTTCCCCC R: AACTCAGACTTCACAGGCTGG	203/229	60	Indel detection
P4	rs668680239 chr14:71400612–71400627	F: CCTGAGAACCAAGCTGTTTGC R: TCTTCTTGACGCCCTTCCCT	272/288	60	Indel detection
P5	rs680105692 chr14:71401507–71401528	F: TCAAACGCCACAGTCAACAC R: CTTGGAAGCATCTCACACGG	275/297	59	Indel detection
P6	rs644524287 chr14:71413164–71413163	F: TGAGCCAGTCCTCAGGTTTAT R: CCCAGACCCACCTACCGTT	212/232	58	Indel detection
P7	rs652252293 chr14:71446770–71446796	F: CAGGTTCACCATTTGCCTGG R: TTTCCAGCGTGTGTAGCAGA	197/224	59	Indel detection
P8	rs660459809 chr14:71451546–71451561	F: CTCACATCGTGGCATTTCTCC R: CAAAGCAAAGCTGAAGGGATG	152/168	58	Indel detection
P9	rs667148743 chr14:71660926–71660927	F: ACAGGAACTGTGTTCCACGAA R: CTAAAGGGAAGGGGAAACTGCT	222/234	59	Indel detection
P10	rs652089744 chr14:71496979–71496996	F: GTCATTGTCTTCGCTTGCTT R: ATGGGTCTGATCCCTGGTTT	229/247	56	Indel detection
*GAPDH*	NC_030812.1	F: AAAGTGGACATCGTCGCCAT R: CCGTTCTCTGCCTTGACTGT	116	60	qRT-PCR
*ASAP1*	NC_030821.1	F: ATGCAGAGGGGGTGGAGTTA R: GCCGTCTGCTTATCCAGGTT	157	60	qRT-PCR
*NF-κB*	NC_030821.1	F: TGGGGATACTGAACAACGCC R: ATCTGTCTCAGGGCCTCCAT	115	60	qRT-PCR
*IL-6*	NC_030821.1	F: TTCAGTCCACTCGCTGTCTC R: TGCTTGGGGTGGTGTCATTC	106	60	qRT-PCR
*TNF-α*	NC_030821.1	F: AACCCATCTACCAGGGAGGG R: AGTAGACCTGCCCAGACTCA	108	60	qRT-PCR
*IFN-γ*	NC_030821.1	F: AGATCCAGCGCAAAGCCATA R: TCTCCGGCCTCGAAAGAGAT	110	60	qRT-PCR
*TGF-β*	NC_030821.1	F: AACAATTCCTGGCGCTACCT R: ACTGAGGCGAAAGCCCTCTA	135	60	qRT-PCR

qRT-PCR, quantitative reverse transcription-polymerase chain reaction.

**Table 2:** Genetic parameters of the P2, P5, and P7 loci of the goat *ASAP1* gene

Loci	Sample	Number	Genotype frequency			Allele frequency		Genetic parameters				HWE

		N	II	ID	DD	I	D	Ho	He	Ne	PIC	p-value
P2	Case	444	0.658 (292)	0.311 (138)	0.031 (14)	0.813	0.187	0.696	0.304	1.437	0.258	p>0.05
	Control	689	0.714 (492)	0.244 (168)	0.042 (29)	0.836	0.164	0.726	0.274	1.378	0.237	p<0.05
	Sum	1,133	0.692 (784)	0.270 (306)	0.038 (43)	0.827	0.173	0.714	0.286	1.401	0.245	p>0.05
P5	Case	406	0.241 (98)	0.483 (196)	0.276 (11)	0.483	0.517	0.501	0.499	1.998	0.354	p>0.05
	Control	648	0.247 (160)	0.494 (320)	0.259 (16)	0.494	0.506	0.500	0.500	2.000	0.353	p>0.05
	Sum	1,054	0.245 (258)	0.490 (516)	0.265 (280)	0.490	0.510	0.500	0.500	1.999	0.375	p>0.05
P7	Case	446	0.514 (229)	0.401 (179)	0.085 (38)	0.714	0.286	0.592	0.408	1.690	0.325	p>0.05
	Control	644	0.419 (270)	0.511 (329)	0.070 (45)	0.675	0.325	0.561	0.439	1.782	0.342	p>0.05
	Sum	1,090	0.458 (499)	0.466 (508)	0.076 (83)	0.691	0.309	0.573	0.427	1.746	0.336	p<0.05

N, number; II, insertion/insertion; ID, insertion/deletion; DD, deletion/deletion; I, insertion; D, deletion; Ho, homozygosity; He, heterozygosity; Ne, effective number of alleles; PIC, polymorphism information; HWE, Hardy-Weinberg equilibrium.

**Table 3:** Distribution of different genotypes and alleles of *ASAP1* gene polymorphic sites in *Brucella* cases and controls

Loci	Genotype/allele	Genotype/allele frequency		χ^2^	p-value

		Case	Control		
P2	II	0.658 (292)	0.714 (492)	6.520	0.038
	ID	0.311 (138)	0.244 (168)		
	DD	0.032 (14)	0.042 (29)		
	I	0.813 (722)	0.835 (1,152)	1.985	0.159
	D	0.187 (166)	0.164 (226)		
P5	II	0.241 (98)	0.247 (160)	0.353	0.838
	ID	0.483 (196)	0.484 (320)		
	DD	0.276 (112)	0.259 (168)		
	I	0.483 (392)	0.494 (640)	0.245	0.621
	D	0.517 (420)	0.506 (656)		
P7	II	0.514 (229)	0.419 (270)	12.703	0.002
	ID	0.401 (179)	0.511 (329)		
	DD	0.085 (38)	0.070 (45)		
	I	0.714 (637)	0.675 (869)	3.837	0.050
	D	0.286 (255)	0.325 (419)		

p<0.05, significant difference.

II, insertion/insertion; ID, insertion/deletion; DD, deletion/deletion.

**Table 4:** Association between age and brucellosis risk

Loci	Age	Frequencies		p-value	OR (95% CI)

		Case	Control		
P2	3	260 (0.586)	420 (0.610)		1.00
	4	140 (0.315)	200 (0.290)	0.577	0.884 (0.574–1.362)
	5	44 (0.099)	69 (0.100)	0.951	0.976 (0.455–2.095)
P5	3	242 (0.596)	395 (0.610)		1.00
	4	124 (0.305)	188 (0.290)	0.723	0.922 (0.588–1.445)
	5	40 (0.099)	65 (0.100)	0.980	0.990 (0.447–2.193)
P7	3	263 (0.590)	393 (0.610)		1.00
	4	138 (0.309)	187 (0.290)	0.657	0.906 (0.586–1.401)
	5	45 (0.101)	64 (0.099)	0.862	0.934 (0.431–2.020)

p<0.05, significant difference.

OR, odds ratio; CI, confidence interval.

**Table 5:** Association between parity and brucellosis risk

Loci	Parity	Frequencies		p-value	OR (95% CI)

		Case	Control		
P2	2	208 (0.468)	336 (0.488)		1.00
	3	150 (0.338)	224 (0.325)	1.000	1.000 (0.679–1.472)
	4	68 (0.153)	101 (147)	1.000	1.000 (0.544–1.840)
	5	18 (0.041)	28 (0.041)	0.978	0.986 (0.370–2.633)
P5	2	194 (0.596)	316 (0.488)		1.00
	3	135 (0.305)	211 (0.326)	0.960	1.010 (0.677–1.509)
	4	61 (0.150)	95 (0.147)	0.981	1.008 (0.533–1.907)
	5	16 (0.039)	26 (0.040)	0.988	1.008 (0.361–2.812)
P7	2	210 (0.471)	314 (0.488)		1.00
	3	150 (0.336)	210 (0.326)	0.987	0.997 (0.675–1.471)
	4	68 (0.152)	94 (0.146)	0.979	1.008 (0.543–1.872)
	5	18 (0.040)	26 (0.040)	0.946	1.035 (0.383–2.795)

p<0.05, significant difference.

OR, odds ratio; CI, confidence interval.

**Table 6:** Relationship between the genetic model of *ASAP1* gene polymorphism sites and the risk of brucellosis

Loci	Genetic models	Genotype/allele	Frequencies		OR (95% CI)	p-value	FDR-adjusted p-value

			Positive	Negative			
P2	Co-dominant	II	0.658 (292)	0.714 (492)	1.00		
		ID	0.311 (138)	0.244 (168)	0.723 (0.553–0.944)	0.017	0.0425
		DD	0.032 (14)	0.042 (29)	1.229 (0.639–2.365)	0.536	0.5360
	Dominant	II	0.658 (292)	0.714 (492)	1.00		
		ID+DD	0.342 (152)	0.286 (197)	0.769 (0.595–0.994)	0.045	0.0750
	Recessive	II+ID	0.968 (430)	0.958 (660)	1.00		
		DD	0.032 (14)	0.042 (29)	1.350 (0.705–2.583)	0.366	0.4575
	Alleles	I	0.813 (722)	0.835 (1,152)	1.00		
		D	0.187 (166)	0.164 (226)	0.853 (0.684–1.064)	0.159	0.2650
P5	Co-dominant	II	0.241 (98)	0.247 (160)	1.00		
		ID	0.483 (196)	0.484 (320)	1.000 (0.735–1.361)	1.000	1.000
		DD	0.276 (112)	0.259 (168)	0.919 (0.649–1.300)	0.632	1.000
	Dominant	II	0.241 (98)	0.247 (160)	1.00		
		ID+DD	0.759 (308)	0.753 (488)	0.970 (0.727–1.296)	0.839	1.000
	Recessive	II+ID	0.724 (294)	0.741 (480)	1.00		
		DD	0.276 (112)	0.259 (168)	0.919 (0.695–1.215)	0.553	1.000
	Alleles	I	0.483 (392)	0.494 (640)	1.00		
		D	0.517 (420)	0.506 (656)	0.957 (0.803–1.140)	0.621	1.000
P7	Co-dominant	II	0.514 (229)	0.419 (270)	1.00		
		ID	0.401 (179)	0.511 (329)	1.559 (1.210–2.008)	0.001	0.005
		DD	0.085 (38)	0.070 (45)	1.004 (0.630–1.601)	0.985	0.985
	Dominant	II	0.514 (229)	0.419 (270)	1.00		
		ID+DD	0.487 (217)	0.581 (374)	1.462 (1.147–1.864)	0.002	0.005
	Recessive	II+ID	0.915 (408)	0.930 (599)	1.00		
		DD	0.085 (38)	0.070 (45)	0.807 (0.514–1.265)	0.349	0.436
	Alleles	I	0.714 (637)	0.675 (869)	1.00		
		D	0.286 (255)	0.325 (419)	1.204 (1.000–1.451)	0.050	0.050

p<0.05, significant difference.

OR, odds ratio; CI, confidence interval; FDR, false discovery rate; II, insertion/insertion; ID, insertion/deletion; DD, deletion/deletion.

**Table 7:** Distribution of *ASAP1* gene locus haplotypes in *Brucella* cases and controls

Haplotypic names	Haplotypic types	Haplotypic frequencies		p-value	OR (95% CI)

		Positive	Negative		
Hap1	I_P2_I_P5_I_P7_	149 (0.188)	263 (0.221)		1.00
Hap2	I_P2_I_P5_D_P7_	140 (0.176)	209 (0.176)	0.263	0.846 (0.631–1.134)
Hap3	I_P2_D_P5_I_P7_	280 (0.354)	376 (0.316)	0.035	0.761 (0.590–0.980)
Hap4	I_P2_D_P5_D_P7_	70 (0.088)	140 (0.118)	0.485	1.133 (0.798–1.608)
Hap5	D_P2_I_P5_I_P7_	94 (0.118)	109 (0.092)	0.016	0.657 (0.467–0.924)
Hap6	D_P2_D_P5_I_P7_	44 (0.056)	62 (0.052)	0.311	0.798 (0.516–1.234)
Hap7	D_P2_I_P5_D_P7_^*^	3 (0.004)	0 (0.000)	-	-
Hap8	D_P2_D_P5_D_P7_^*^	13 (0.016)	29 (0.024)	-	-

p<0.05, significant difference.

* All frequencies below 0.03 were ignored in this analysis.

OR, odds ratio; CI, confidence interval.

## Data Availability

Upon reasonable request, the datasets of this study can be available from the corresponding author.

## SUPPLEMENTARY MATERIAL

**Supplement 1**. Differential binding of transcription factors between II and DD genotypes at the P2 and P7 loci of the *ASAP1* gene.

**Supplement 2**. Prediction of putative transcription factor binding sites at the P2 and P7 loci of the *ASAP1* gene.

**Supplement 3**. Bioinformatics analysis of the goat *ASAP1* gene.
