# Supplementary material for: ASAP1 gene InDel variants are associated with enhanced goat resistance against Brucella infection
Source: Anim Biosci. 2026 Mar 11;39(7):250722. doi: 10.5713/ab.250722 (PMC13353162; doi:10.5713/ab.250722)
Supplement: Supplementary file 1 [file ab-250722-Supplementary-1.pdf]

Supplement 1. Differential binding of transcription factors between II and DD genotypes at the P2 and P7 loci of the *ASAP1* gene

**Loci**	**Sites**	**Transcription factors**		**Differential transcription factors**
		**Insertion**	**Deletion**	
P2	rs655531471 chr14:71407951 -71407952	C/EBPalp、MCM1、HNF-1、Ftz	C/EBPalp、P1-site、HNF-1、Ftz	MCM1、P1-site
`P7	rs652252293 chr14:71446770 -71446796	SP1、Egr-1、CREB、CPEbind、AP-2alph	SP1、Egr-1、AP-2alph	CREB、CPEbind
