# Supplementary material for: ASAP1 gene InDel variants are associated with enhanced goat resistance against Brucella infection
Source: Anim Biosci. 2026 Mar 11;39(7):250722. doi: 10.5713/ab.250722 (PMC13353162; doi:10.5713/ab.250722)
Supplement: Supplementary file 2 [file ab-250722-Supplementary-2.pdf]

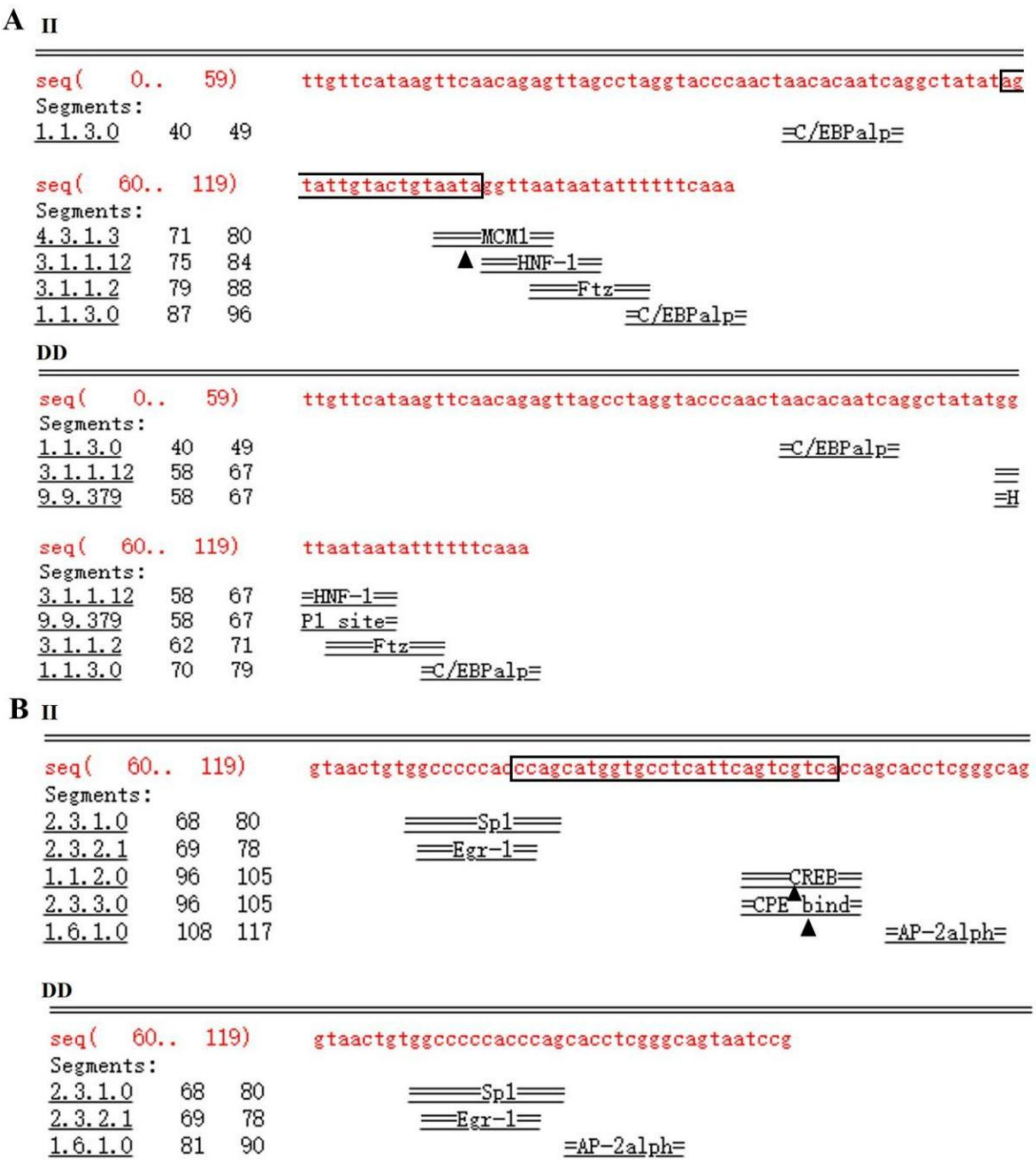

Supplement 2. Prediction of putative transcription factor binding sites at the P2 and P7 loci of the *ASAP1* gene

(A) Predicted binding profile for the II and DD genotypes at the P2 locus;

(B) Predicted binding profile for the II and DD genotypes at the P7 locus.
