# Supplementary material for: ASAP1 gene InDel variants are associated with enhanced goat resistance against Brucella infection
Source: Anim Biosci. 2026 Mar 11;39(7):250722. doi: 10.5713/ab.250722 (PMC13353162; doi:10.5713/ab.250722)
Supplement: Supplementary file 3 [file ab-250722-Supplementary-3.pdf]

A

Percent identity / %

Divergence / %

	1	2	3	4	5	6		
**1**		98.7	96.3	89.0	71.4	86.4	**1**	***Capra hircus(XM_018058570.1)***
**2**	1.4		96.5	88.7	71.1	86.1	**2**	***Ovis aries(XM_027972897.3)***
**3**	3.8	3.6		89.0	71.0	86.0	**3**	***Bos taurus (NM_175821.3)***
**4**	12.0	12.4	12		71.1	85.8	**4**	***Sus scrofa(XM_021088909.1)***
**5**	36.9	37.3	37.5	37.4		72.9	**5**	***Gallus gallus(XM_025148177.3)***
**6**	15.2	15.5	15.7	15.8	34.3		**6**	***Homo sapiens(NM_001247996.2)***
	**1**	**2**	**3**	**4**	**5**	**6**		

B

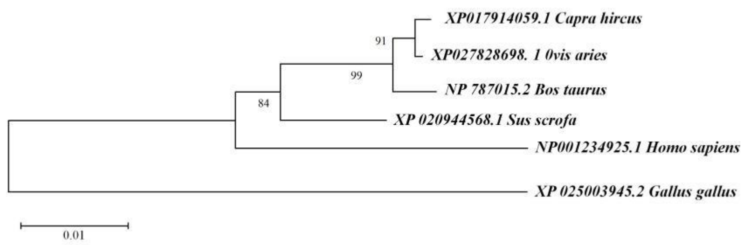

Supplement 3. Bioinformatics analysis of the goat *ASAP1* gene

(A) Nucleic acid sequence homology analysis of the *ASAP1* gene;

(B) Phylogenetic tree of the *ASAP1* gene in different animal species.
